# Associations of serum lead with colorectal cancer: data from NHANES 1999-2020

**DOI:** 10.7150/jca.117774

**Published:** 2025-10-01

**Authors:** Lin Zhong, Yuanhong Peng, Lina Luo, Luji Huang, Fu Cheng, Yan Lu, Yongle Ju, Manzhao Ouyang

**Affiliations:** 1 Department of Gastrointestinal Surgery, The Eighth Affiliated Hospital, Southern Medical University (The First People's Hospital of Shunde, Foshan), Shunde, Foshan, Guangdong Province, 528300, China.; 2 The Second School of Clinical Medicine, Southern Medical University, Guangzhou, Guangdong Province, 510080, China.; 3 Jianhe People's Hospital, Qiandongnan Miao and Dong Autonomous Prefecture, Guizhou Province, 556400, China.; 4 GCP Center, The Eighth Affiliated Hospital, Southern Medical University (The First People's Hospital of Shunde, Foshan), Shunde, Foshan, Guangdong Province, 528300, China.

**Keywords:** Serum lead, Colorectal cancer, NHANES

## Abstract

**Background:** Exposure to lead, a harmful heavy metal, is one of the risk factors for the development of Colorectal cancer (CRC). However, limited information is available on the impact of serum lead on the incidence of CRC. Therefore, this study utilized data from the National Health and Nutrition Examination Survey (NHANES) to explore the relationship between serum lead and CRC.

**Methods:** A total of 32,894 American adults from the 1999-2020 NHANES cycles were included in this study, among whom 225 reported having CRC. Additionally, we also collected data on 3,024 other cancer patients from the same period. Weighted logistic regression analysis was used to calculate the odds ratio (OR) and 95% confidence interval (CI) for the risk of CRC associated with serum lead, with adjustments for potential confounding factors. Restricted cubic spline (RCS) analysis was conducted to examine the dose-response relationship between serum lead and the risk of CRC. Concurrently, we performed propensity score matching (PSM) analysis and validated our conclusions through ICP-MS quantification in clinical tissue specimens.

**Results:** This study revealed that patients diagnosed with CRC exhibited significantly elevated serum lead levels in comparison to both the general population and other cancer cohorts. Weighted logistic regression analysis showed a significant positive correlation between serum lead and the risk of CRC with or without adjusting for sociodemographic variables, BMI, diabetes, hypertension, and other covariates. The RCS model detected a dose-response relationship. Subgroup analysis indicated that the association between serum lead and CRC was similar across different sociodemographic characteristics, health behaviors, and comorbidities. However, the risk of CRC increased with higher serum lead levels among individuals aged ≥45, males, whites, BMI ≥24, alcohol users, smokers, and patients with diabetes. Despite the lack of statistically significant differences in lead levels after PSM analysis—potentially attributable to cohort size variations—our ultimate ICP-MS quantification of clinical tissues revealed markedly elevated lead concentrations in CRC specimens (*p* <0.0001).

**Conclusion:** This cross-sectional study indicates a significant positive correlation between serum lead and the risk of CRC. Further prospective studies are needed to confirm these findings and elucidate the underlying mechanisms.

## 1. Introduction

Colorectal cancer (CRC) is the third most common malignant tumor globally and one of the leading causes of cancer-related deaths, posing a significant threat to public health^1^. Due to the lack of effective early diagnostic methods, most patients seek medical attention only when symptoms arise, by which time the disease is often in advanced stages. This limits treatment options and contributes to poor patient prognosis^2^. Therefore, it is essential to explore the mechanisms underlying its development to establish effective strategies for prevention, early diagnosis, and treatment, ultimately improving patient outcomes. However, the development and progression of CRC is a complex process influenced by various factors, including diet, environment, lifestyle, and genetics.

With industrial development and changes in dietary patterns, exposure to heavy metal ions (such as mercury, lead, cadmium, and arsenic) has increased, leading to a higher cancer risk. Studies have shown that 80% of cancer cases are related to environmental factors, including trace elements and heavy metals^3^. Heavy metal can enter the body through the digestive, respiratory, and skin systems, accumulating in various organs^4^. Once heavy metal levels reach a certain threshold, they can trigger a series of pathophysiological reactions, such as oxidative stress and apoptosis, which are associated with an increased risk of tumors, cardiovascular diseases, diabetes, developmental disorders, and neurological diseases^5^,^6^. To date, most studies have roughly estimated heavy metal exposure through occupational exposure and dietary intake^7^, but these methods lack precision and incur high research costs. Moreover, once absorbed, heavy metal convert into various forms and distribute throughout the body, making it difficult to accurately determine their exposure levels and their association with diseases.

Notably, an increasing number of researchers suggest using blood levels of heavy metals as a more accurate surrogate indicator of exposure levels^8^. Lead, one of the most abundant harmful heavy metals, is implicated in the development of various tumors. Reports indicate that the content of lead in CRC tissues is significantly higher than in healthy tissues^9^. However, there is insufficient evidence on the correlation between serum lead levels and CRC risk in the general population. This study analyzed data from the National Health and Nutrition Examination Survey (NHANES) to investigate the relationship between blood lead and CRC, providing further evidence for the critical role of lead in the development and progression of CRC.

## 2. Materials and Methods

### 2.1 Data collection and participants

All data for this study were sourced from the NHANES, a program designed to evaluate the health and nutritional status of adults and children in the United States. Detailed information can be accessed at *https://www.cdc.gov/nchs/nhanes/index.htm*. The NHANES protocol was approved by the National Center for Health Statistics' Institutional Review Board of the Centers for Disease Control and Prevention, with informed consent obtained from all participants^10^,^11^. This open-access, freely available database comprises five modules (Demographics Data, Dietary Data, Examination Data, Laboratory Data, and Questionnaire Data), from which we extracted demographic variables (age, gender, education) from Module “Demographics Data”, serum lead concentrations from Module “Laboratory Data”, and lifestyle/clinical factors (smoking, alcohol consumption, hypertension, diabetes) from Module “Questionnaire Data”, subsequently importing all data into Rstudio for merging, pruning records with missing information, and preparing for further analyses. This study utilized NHANES data spanning from 1999 to 2020. The primary exclusion criteria included missing serum lead data, inability to determine CRC status, absent weight information, and incomplete covariates (such as age, gender, race, BMI, etc.).

### 2.2 Diagnosis of colorectal cancer and other cancers

This study identified cases of CRC using medical condition records from the NHANES Questionnaire. Participants were asked to respond to the questionnaire item "Ever told you had cancer or malignancy?". If they answered yes, they were further asked to specify the type of cancer. Interviewers coded the reported cancer types, which were then summarized into categories^12^. Based on responses to the MCQ220 questionnaire, individuals answering "YES" were identified as cancer patients. Subsequent classification of cancer type was determined by MCQ230a responses. After excluding cases with missing values, this study defined the CRC patient cohort as those reporting "Colon", "Rectum (rectal)", and “colorectal cancer”. All other cancer patients were aggregated into a single comparison group for subsequent analyses.

### 2.3 Measurement of serum lead

During the sample dilution process, a small amount of whole blood is extracted from a larger patient specimen to ensure an even distribution of cellular components. Prior to blood collection, an anticoagulant like EDTA is added to the collection tube to prevent clot formation, which could otherwise disrupt the uniformity of cellular material in the sample. Analysts inspect each sample for the presence of clots or microclots (tiny clots) both before and during sample preparation. Samples containing clots cannot be analyzed using this method due to potential issues with uniformity, rendering all results from such samples as "unreportable". The sample undergoes dilution with a diluent containing tetramethylammonium hydroxide (0.4% v/v), Triton X-100™ (0.05%), and ammonium pyrrolidinedithiocarbamate (APDC). The resulting liquid sample is then introduced into the mass spectrometer via an inductively coupled plasma ionization source for detection.

Weigh out 1g of fresh colon tissue or CRC tumor tissue. Rinse it three times with pure water, then add 1mL of ultrapure water and 2mL of nitric acid. Transfer the mixture to a super microwave digestion system for digestion. Subsequently, dilute the digest to 25mL with water, filter to remove residues, and proceed to instrumental analysis.

### 2.4 Covariates

The covariates considered in this study encompass sociodemographic, behavioral, and health characteristics considered as potential confounding factors. Sociodemographic variables include age (<45 years and ≥45 years), gender (female and male), race/ethnicity (non-Hispanic Black, non-Hispanic White, Mexican American, or other races), educational level (Below high school, High school, or Above high school), and poverty income ratio. Behavioral characteristics include smoking status (smoker or non-smoker) and alcohol consumption (non-drinker or drinker). Health factors include body mass index (BMI <24 or ≥24), hypertension (no or yes), and diabetes (no or yes).

### 2.5 Statistical analysis

The basic characteristics of participants were presented using mean ± standard error or frequency and corresponding weighted percentages. Survey-weighted logistic regression was employed to investigate the association between serum lead levels and the likelihood of CRC. Three hierarchical models were constructed with progressive adjustments for potential confounding factors. Model I conducted an independent analysis of lead ion levels. Model II adjusted for sociodemographic characteristics, including age, gender, race/ethnicity, education level, household income, and BMI. Model III further accounted for health behaviors and comorbidities by incorporating smoking, alcohol consumption, hypertension, and diabetes into the factors considered in Model II. Restricted cubic spline regression was applied to further elucidate the relationship between serum lead levels and CRC risk.

To minimize selection bias between CRC patients and healthy individuals, this study employed propensity score matching (PSM) to adjust for confounding factors. PSM utilizes logistic regression modeling where the intervention factor serves as the dependent variable, and all observed non-study factors act as independent variables. This approach estimates the probability of an individual being diagnosed with CRC given their covariates, thereby generating a propensity score reflecting their likelihood of CRC diagnosis. All statistical analyses were performed using R software, with *p* < 0.05 considered statistically significant.

## 3. Results

### 3.1 Baseline characteristics

**Figure 1:**
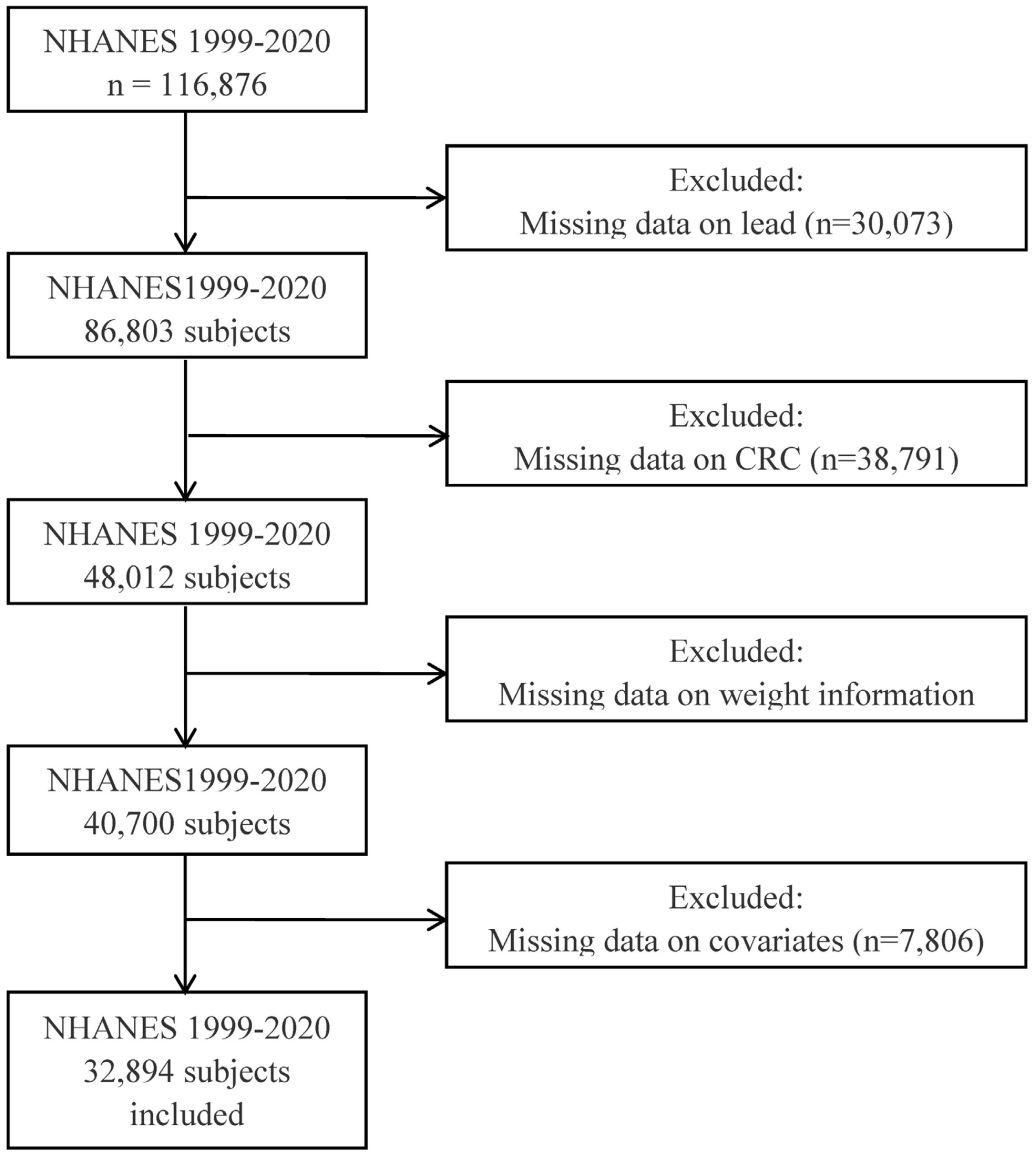
The flow chart of the study.

As shown in Figure 1, this study included data from NHANES from 1999 to 2020, comprising a total of 116,876 participants. Following exclusions of data lacking serum lead ion information, indeterminate CRC status, missing weight data, and essential covariates, 32,894 participants were ultimately included. Among these, 32,669 were classified in the non-tumor group, while 225 were diagnosed with CRC. Furthermore, the study compiled data from 3,024 participants diagnosed with other types of tumors for further investigation.

### 3.2 Weighted logistic regression analysis on the association between serum lead and colorectal cancer

Table 2 presents the results of weighted logistic regression analyses examining the association between serum lead and CRC. First, we constructed a weighted logistic regression model comparing the normal population with CRC patients. Model I, which did not adjust for any covariates, shows a significant positive risk correlation between serum lead levels and CRC (OR = 1.09, *p* < 0.0001). Upon adjusting for covariates including age, gender, race, PIR, BMI, and education level in Model II, this positive correlation remained (OR = 1.07, *p* < 0.001). Model III, which additionally included covariates related to health behaviors and comorbidities, also indicated a significant positive correlation between serum lead levels and CRC risk (OR = 1.08, *p* < 0.0001).

Next, we conducted a weighted logistic regression analysis between CRC and other tumor groups. Remarkably, a significant positive risk correlation between serum lead levels and CRC was still observed. In these analyses, Model I did not adjust for any covariates, Model II adjusted for covariates including age, gender, race, PIR, BMI, and education level, and Model II adjusted for age, gender, race, PIR, BMI, education level, diabetes, hypertension, smoking, and alcohol consumption.

### 3.3 Dose-response relationships between blood lead and colorectal cancer

**Figure 2:**
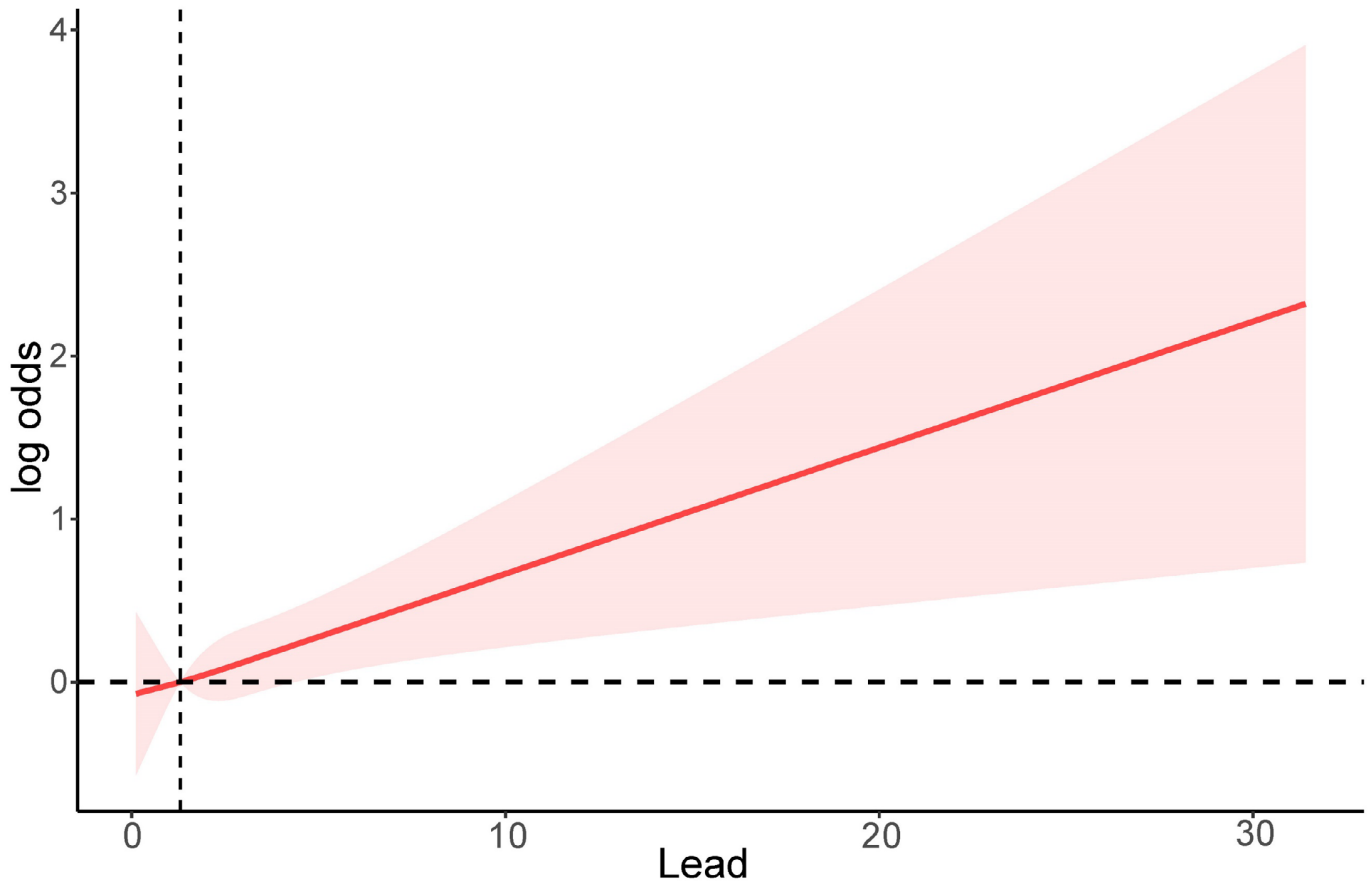
A restricted cubic spline plot of the association between serum lead levels and the risk of CRC. The solid line represents the odds ratio (OR) for CRC at different lead levels, and the shaded area represents the 95% confidence interval.

To explore the dose-response relationship between serum lead and the risk of CRC, we performed a restricted cubic spline analysis based on logistic regression. As shown in Figure 2, the RCS model indicates that higher serum lead ion levels are associated with an increased risk of CRC, with a non-linear p-value of 0.0045.

### 3.4 Stratified analyses

**Figure 3:**
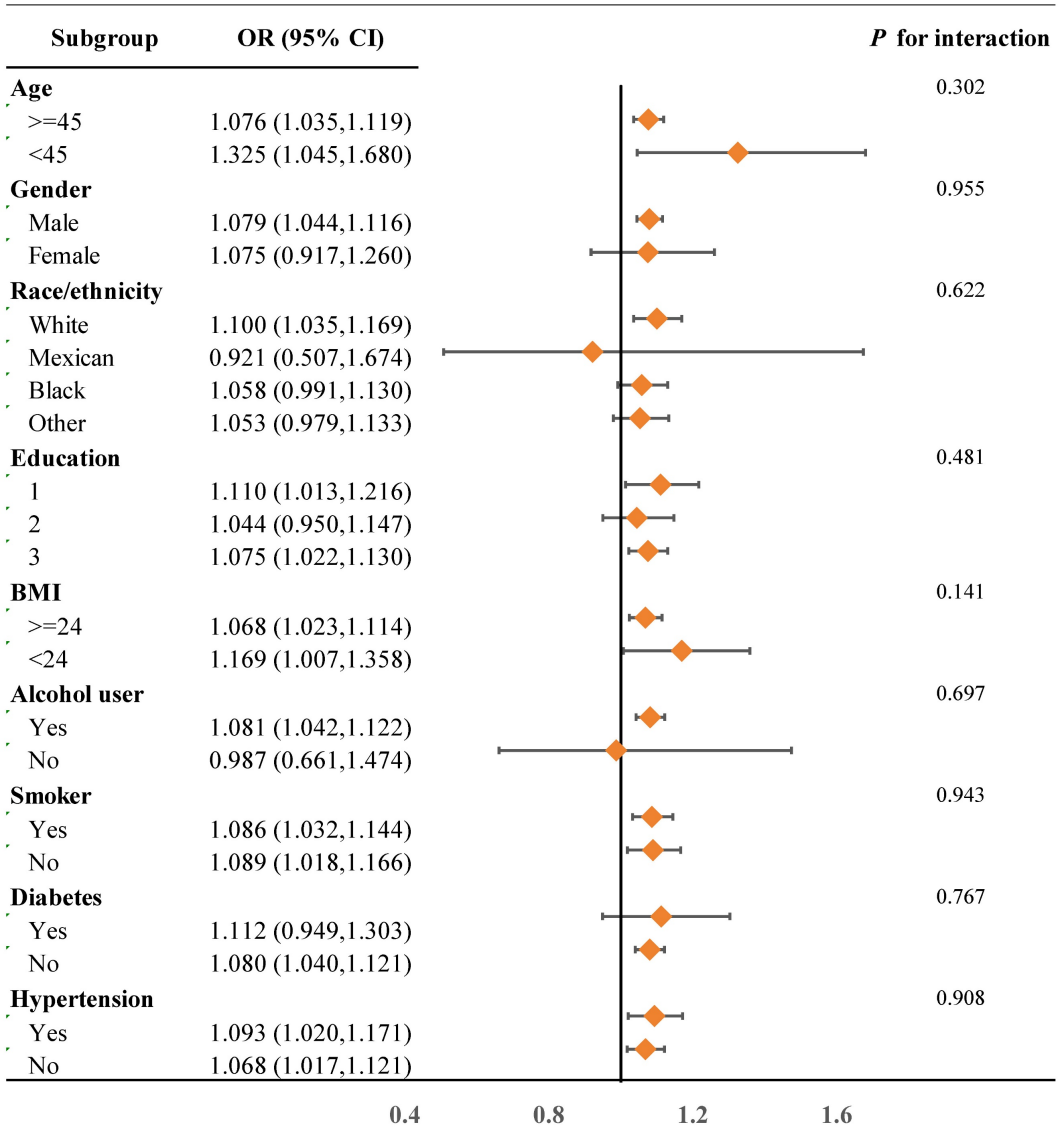
Subgroup analysis of serum lead levels and CRC risk stratified by baseline characteristics. Education: 1. Below high school; 2. High school; 3. Above high school.

Further stratified analyses were conducted to examine the relationship between serum lead levels and sociodemographic characteristics, health behaviors, and comorbidities. As shown in Figure 3, overall, the association between serum lead levels and the risk of CRC is similar across different sociodemographic characteristics, health behaviors, and comorbidities. However, among individuals aged ≥45, males, white individuals, BMI ≥24, alcohol users, smokers, and individuals with diabetes, the risk of CRC increases with higher serum lead levels.

### 3.5 PSM analysis

To minimize selection bias between CRC patients and healthy controls, this study employed propensity score matching (PSM) to address confounding factors. Key findings after PSM implementation: (1) Serum lead levels were significantly elevated in CRC patients versus controls (*p* = 0.07) (Table 3), though this marginal significance may reflect baseline intergroup imbalances; (2) Subsequent survey-weighted logistic regression—both unadjusted and adjusted for confounders—consistently established elevated serum lead as an independent risk factor for CRC (Table 4). These outcomes align with prior evidence, reinforcing methodological robustness.

### 3.6 Lead levels in clinical tissues

**Figure 4:**
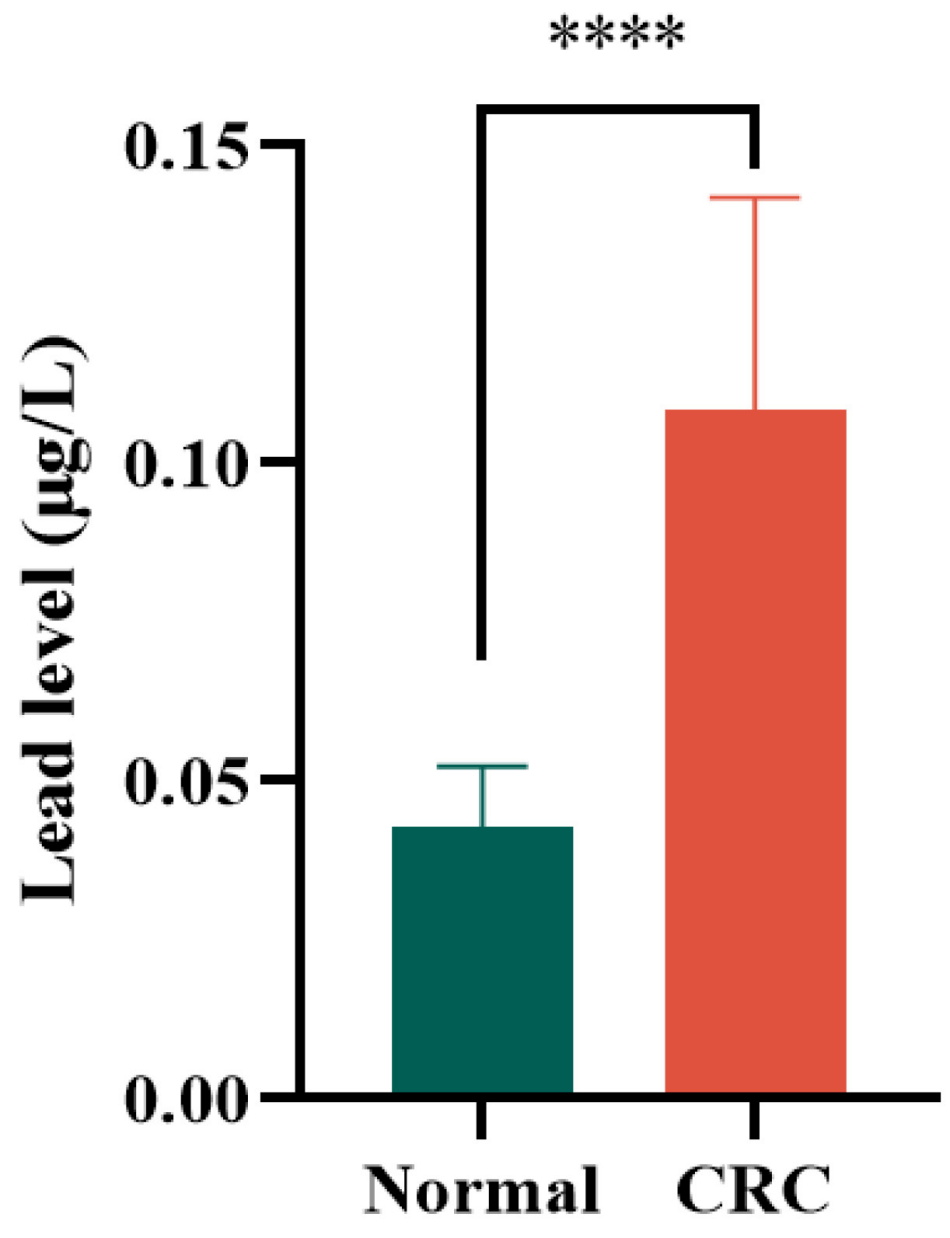
Results of ICP-MS detection of lead levels in colon tissues from 8 healthy controls and tumor tissues from 12 CRC patients.

All findings presented herein were derived from the NHANES database. To provide orthogonal validation of these results, we conducted ICP-MS analysis comparing lead distribution in colon tissues from 8 healthy controls versus tumor tissues from 12 CRC patients. The data revealed significantly elevated lead levels in CRC specimens relative to healthy tissues (mean 0.04286 vs 0.1085 μg/L; *p* < 0.0001) (Figure 4). These converging lines of evidence suggest that elevated lead exposure may represent a potential independent risk factor for CRC development.

## 4. Discussion

This project is a cross-sectional study utilizing the NHANES database to investigate the relationship between serum heavy metal ions and CRC. Upon comparing the baseline characteristics of CRC patients with those of the general population, we observed significantly higher serum lead ion levels among CRC patients. Furthermore, there were notable statistical differences between the groups in terms of age, race, BMI, smoking status, hypertension, and diabetes. Consequently, these variables were included as confounding factors in our analytical model for subsequent analyses. Importantly, even after adjusting for these confounders, the difference in serum lead levels between the normal population and CRC patients remained significant, consistent with our initial findings. The RCS curve analysis illustrated varying dose-response relationships between serum lead and CRC risk. These results suggest that elevated serum lead levels constitute a risk factor for CRC. Lead is a widely dispersed and abundant toxic heavy metal globally, classified as a Group 2B carcinogen by the International Agency for Research on Cancer of the World Health Organization in 2017. Routes of human exposure to lead include inhalation of automobile exhaust, drinking water contaminated with industrial wastewater, ingestion of lead-contaminated food, and direct skin contact^13^,^14^. Due to the body's limited capacity to eliminate lead^15^, absorbed lead accumulates in organs such as the liver, kidneys, and bones as it circulates with the blood, causing acute or chronic negative health effects. Research has demonstrated that lead can adversely affect the reproductive, immune, liver, and gastrointestinal systems, disrupting normal biochemical and physiological processes in the body^14^.

Lead exposure is known to increase the risk of cancer. However, current assessments primarily rely on rough estimates derived from occupational exposure and dietary surveys. In reality, lead exists in various compounds and undergoes transformations within the human body, and accumulates in different organs. This variability complicates efforts to accurately establish the relationship between lead exposure and the onset of disease. Recently, blood lead levels have gained recognition as a crucial indicator of exposure among researchers. Studies have highlighted abnormal distributions of metal ions in tumors. For instance, research involving 2,587 breast cancer cases indicated that elevated lead levels were linked to risk of postmenopausal breast cancer (OR = 1.1)^16^. Elevated blood lead levels have also been observed in other cancers such as lung, ovarian, oral, and gastrointestinal tumors, potentially influencing tumor pathogenesis^17^-^20^. Consistently, our study found significantly higher blood lead levels among CRC patients compared to the general population. Interestingly, when comparing blood lead levels between CRC and other tumor patients, we unexpectedly discovered notably higher levels in CRC patients, suggesting that lead may play a pivotal role in CRC development and could serve as a potential serum biomarker. More importantly, when we measured lead ion levels in healthy colon tissue and colorectal cancer tissue, we found that levels in cancerous tissue were significantly higher—approximately 2.5 times higher than in normal intestinal tissue. This strongly suggests a close relationship between lead ion levels and colorectal cancer.

The gastrointestinal tract, being the primary site for food digestion and absorption, undergoes significant metabolic changes when contaminated food with excessive lead is ingested. Lead can damage the intestinal barrier function and disrupt the gut microbiota ecosystem, leading to intestinal lesions and potentially inducing the development of gastrointestinal tumors^21^. However, research on how lead induces the occurrence and development of CRC is still very limited. Several potential carcinogenic mechanisms of lead have been reported. First, lead increases reactive oxygen species (ROS) levels by reducing the activity of antioxidant enzymes and activating the Nrf2/Keap1 signaling pathway, leading to cellular oxidative stress and DNA damage^22^-^25^; Secondly, lead inhibits DNA repair and alters protein structures, resulting in uncontrolled cell growth and disrupted cell signal transduction^26^,^27^; Furthermore, lead can induce the production of interleukin-8 (IL-8), promoting tumor angiogenesis and invasion^28^,^29^, and cause imbalances in oncogenes and tumor suppressor genes by altering DNA methylation levels^30^,^31^. In summary, lead is widely regarded as a significant risk factor for CRC. However, its potential roles and mechanisms in the occurrence and development of CRC remain to be further explored.

NHANES is a nationally representative database that includes comprehensive data on demographics, diet, examinations, laboratory tests, and questionnaires. Researchers can use the sample weights provided by NHANES to generalize their findings to the national population, offering valuable insights into lifestyle, diet, and other behaviors. This study utilized NHANES data to construct a logistic regression model, adjusting for key confounding factors. The results revealed that serum lead levels are an independent risk factor for CRC, providing a reliable preliminary basis for the prevention and early diagnosis of this disease. However, there are some limitations in this study. First, as a cross-sectional study based on NHANES data, it only reflects the serum lead levels of individuals at the time of testing, rather than their long-term exposure. Second, due to missing information, we cannot completely rule out the influence of important confounding factors such as environmental exposure and genetic factors. Additionally, while we observed significantly elevated serum lead levels in CRC patients, it is difficult to infer a causal relationship between serum lead and CRC from this cross-sectional study. Therefore, further prospective studies are needed to clarify the relationship between serum lead levels and CRC. Moreover, deeper mechanistic studies are required to elucidate the critical role of lead in the occurrence and development of CRC.

## 5. Conclusion

In conclusion, our analysis of NHANES data and clinical tissues revealed that serum lead levels are significantly elevated in CRC patients compared to both the general population and patients with other tumors. This finding provides a solid theoretical foundation for the prevention and early diagnosis of CRC. Further research is needed to explore the crucial role of lead in the pathogenesis of CRC.

## Figures and Tables

**Table 1:** Characteristics of participants in the NHANES 1999-2020 cycles

Characteristic	NHANES participants from 1999 to 2020 (n=32894)			NHANES Tumor participants from 1999 to 2020 (n=3249)		
	Non-Tumor (n=32669)	CRC (n=225)	*P* value	Other Tumor (n=3024)	CRC (n=225)	*P* value
**Lead(umol/L)**	1.589 (0.017)	2.265 (0.155)	< 0.0001	1.832 (0.032)	2.265 (0.155)	0.006
PIR	3.025 (0.027)	2.895 (0.124)	0.274	3.292 (0.047)	2.895 (0.124)	0.002
Age group			< 0.0001			0.015
<45	15193 (51.267)	5 (3.954)		326 (14.116)	5 (3.954)	
>=45	17476 (48.733)	220 (96.046)		2698 (85.884)	220 (96.046)	
Gender			0.456			0.013
Female	16664 (50.620)	105 (53.872)		382 (4.730)	42 (8.426)	
Male	16005 (49.380)	120 (46.128)		203 (1.938)	13 (1.464)	
Race/ethnicity			< 0.0001	224 (4.582)	20 (5.739)	
Black	6702 (10.736)	42 (8.426)		2215 (88.750)	150 (84.371)	
Mexican	6050 (8.095)	13 (1.464)				0.328
Other^a^	5184 (11.820)	20 (5.739)		1601 (58.355)	105 (53.872)	
White	14733 (69.349)	150 (84.371)		1423 (41.645)	120 (46.128)	
Educational^b^			0.195			0.059
1	8570 (16.436)	75 (22.187)		659 (14.459)	75 (22.187)	
2	7563 (24.233)	51 (22.062)		709 (22.940)	51 (22.062)	
3	16536 (59.331)	99 (55.751)		1656 (62.601)	99 (55.751)	
BMI			0.011			0.024
<24	7435 (24.392)	36 (16.039)		676 (23.502)	36 (16.039)	
>=24	25234 (75.608)	189 (83.961)		2348 (76.498)	189 (83.961)	
Smoker			0.005			0.357
No	17799 (54.186)	89 (40.068)		1325 (44.775)	89 (40.068)	
Yes	14870 (45.814)	136 (59.932)		1699 (55.225)	136 (59.932)	
Alcohol user			0.947			0.827
No	4588 (10.889)	27 (11.065)		400 (10.486)	27 (11.065)	
Yes	28081 (89.111)	198 (88.935)		2624(89.514)	198(88.935)	
Hypertension			< 0.0001			0.003
No	22134 (71.983)	70 (36.491)		1384 (51.566)	70 (36.491)	
Yes	10535 (28.017)	155 (63.509)		1640 (48.434)	155 (63.509)	
Diabetes			< 0.0001			0.073
No	29127 (92.370)	173 (79.664)		2493 (85.288)	173 (79.664)	
Yes	3542 (7.630)	52 (20.336)		531 (14.712)	52 (20.336)	

NHANES: National Health and Nutrition Examination Survey; CRC: Colorectal cancer; PIR: Poverty income ratio; BMI: body mass index. ^a^ Included multiracial participants. ^b^ 1: Below high school; 2: High school; 3: Above high school.

**Table 2:** Association of serum lead with CRC among participants in the NHANES 1999-2020 cycles

	Model I		Model II		Model III	
		P value		P value		P value
	OR (95% CI)		OR (95% CI)		OR (95% CI)	
**Non-Tumor vs CRC**	1.09 (1.06, 1.12)	<0.0001	1.07 (1.03, 1.12)	<0.001	1.08 (1.04, 1.12)	<0.0001
**Other vs CRC**	1.19 (1.09, 1.30)	<0.001	1.14 (1.03, 1.26)	0.02	1.14 (1.03	0.01

**Model I:** No modification variables; **Model II:** Adjusts for PIR, age, gender, race, education, and BMI; **Model III:** Adjusts PIR, age, gender, race, education, BMI, smoker, alcohol user, hypertension, diabetes.

**Table 3:** Characteristics of participants in the NHANES 1999-2020 cycles after PSM analysis

Characteristic	Non-Tumor (n=32669)	CRC (n=225)	*P* value
**Lead**	1.965(0.065)	2.265(0.155)	0.078
PIR	3.076(0.085)	2.895(0.124)	0.184
Age group			0.809
<45	18(3.512)	5(3.954)	
>=45	657(96.488)	220(96.046)	
Gender			0.509
Female	296(47.590)	105(53.872)	
Male	379(52.410)	120(46.128)	
Race/ethnicity			0.877
Black	121(6.832)	42(8.426)	
Mexican	44(1.783)	13(1.464)	
Other	55(4.566)	20(5.739)	
White	455(86.820)	150(84.371)	
Education			0.871
1	212(22.941)	75(22.187)	
2	153(23.831)	51(22.062)	
3	310(53.227)	99(55.751)	
BMI			0.918
<24	106(18.390)	36(16.039)	
>=24	569(81.610)	189(83.961)	
Smoker			0.287
No	231(33.186)	89(40.068)	
Yes	444(66.814)	136(59.932)	
Alcohol user			0.368
No	66( 8.041)	27(11.065)	
Yes	609(91.959)	198(88.935)	
Hypertension			0.975
No	209(37.731)	70(36.491)	
Yes	466(62.269)	155(63.509)	
Diabetes			0.711
No	510(80.626)	173(79.664)	
Yes	165(19.374)	52(20.336)	

CRC: Colorectal cancer; PIR: Poverty income ratio; BMI: body mass index. ^a^ Included multiracial participants. ^b^ 1: Below high school; 2: High school; 3: Above high school.

**Table 4:** Association of serum lead with CRC among participants in the NHANES 1999-2020 cycles after PSM analysis

	Model I		Model II		Model III	
		*P* value		*P* value		*P* value
	OR (95% CI)		OR (95% CI)		OR (95% CI)	
**Non-Tumor vs CRC**	1.12 (1.00, 1.25)	0.04	1.17 (1.05, 1.31)	0.01	1.19 (1.07, 1.33)	0.002

**Model I:** No modification variables; **Model II:** Adjusts for PIR, age, gender, race, education, and BMI; **Model III:** Adjusts PIR, age, gender, race, education, BMI, smoker, alcohol user, hypertension, diabetes.
